# Gamified Web-Delivered Attentional Bias Modification Training for Adults With Chronic Pain: Protocol for a Randomized, Double-blind, Placebo-Controlled Trial

**DOI:** 10.2196/32359

**Published:** 2022-01-27

**Authors:** Julie F Vermeir, Melanie J White, Daniel Johnson, Geert Crombez, Dimitri M L Van Ryckeghem

**Affiliations:** 1 School of Psychology and Counselling, Faculty of Health Queensland University of Technology Brisbane Australia; 2 Faculty of Science Queensland University of Technology Brisbane Australia; 3 Department of Experimental Clinical and Health Psychology Ghent University Ghent Belgium; 4 Department of Clinical Psychological Science Maastricht University Maastricht Netherlands; 5 Department of Behavioural and Cognitive Sciences University of Luxembourg Esch-sur-Alzette Luxembourg

**Keywords:** chronic pain, cognition, attentional bias, gamification, motivation, randomized controlled trial, web-based intervention, pain management, digital intervention, digital health

## Abstract

**Background:**

To date, research has found variable success in using attentional bias modification training (ABMT) procedures in pain samples. Several factors could contribute to these mixed findings, including boredom and low motivation. Indeed, training paradigms are repetitive, which can lead to disengagement and high dropout rates. A potential approach to overcoming some of these barriers is to attempt to increase motivation and engagement through gamification (ie, the use of game elements) of this procedure. To date, research has yet to explore the gamified format of ABMT for chronic pain and its potential for the transfer of benefits.

**Objective:**

The aim of this study is to investigate the effects of a gamified web-delivered ABMT intervention in a sample of adults with chronic pain via a randomized, double-blind, placebo-controlled trial.

**Methods:**

A total of 120 adults with chronic musculoskeletal pain, recruited from clinical (hospital outpatient waiting list) and nonclinical (wider community) settings, will be included in this randomized, double-blind, placebo-controlled, 3-arm trial. Participants will be randomly assigned to complete 6 web-based sessions of dot-probe nongamified sham control ABMT, nongamified standard ABMT, or gamified ABMT across a period of 3 weeks. Active ABMT conditions will aim to train attention away from pain-relevant words. Participant outcomes will be assessed at pretraining, during training, immediately after training, and at the 1-month follow-up. Primary outcomes include pain intensity, pain interference, and behavioral and self-reported engagement. Secondary outcomes include attentional bias for pain, anxiety, depression, interpretation bias for pain, and perceived improvement.

**Results:**

The ethical aspects of this research project have been approved by the human research ethics committees of the Royal Brisbane and Women’s Hospital (HREC/2020/QRBW/61743) and Queensland University of Technology (2000000395). Study recruitment commenced in August 2021 and is ongoing. Data collection and analysis are expected to be concluded by October 2022 and January 2023, respectively.

**Conclusions:**

This trial will be the first to evaluate the effects of gamification techniques in a pain ABMT intervention. The findings will provide important information on the potential therapeutic benefits of gamified pain ABMT programs, shed light on the motivational influences of certain game elements in the context of pain, and advance our understanding of chronic pain.

**Trial Registration:**

Australian New Zealand Clinical Trials Registry ACTRN12620000803998; https://anzctr.org.au/ACTRN12620000803998.aspx

**International Registered Report Identifier (IRRID):**

PRR1-10.2196/32359

## Introduction

### Background

Attention in individuals with chronic pain is often biased toward pain-related information (ie, word or picture stimuli) [1-3]. Such findings have led researchers to investigate whether these attentional biases can be modified with an attentional bias modification training (ABMT) procedure and whether this modification leads to changes in pain intensity and associated pain-related health outcomes [4]. ABMT protocols typically use a modified dot-probe task [5] to train participants to disengage from pain-related information and redirect attention to the competing neutral cues. To date, research has found variable success in using ABMT in pain samples (see the study by Van Ryckeghem et al [6] for an overview). Specifically, in the context of chronic pain, some studies have indicated that ABMT can be effective in improving pain-related outcomes (eg, pain intensity and pain-related disability) [4,7,8], whereas others have failed to replicate these positive effects [9,10].

Several factors could contribute to these mixed findings, including boredom and low motivation. Qualitative studies have indicated that participants experience dot-probe tasks as monotonous, repetitive, and boring [11,12]. Indeed, ABMT procedures require systematic repetition of numerous trials over multiple sessions across several weeks [4,7-10] and typically include a basic layout (ie, stimuli are presented on a plain background), which may make training sessions unappealing. The monotonous nature of such tasks could lead to (temporal) disengagement, low motivation, and high dropout rates, which in turn may compromise intervention efficacy. A potential approach to overcoming some of these barriers is to attempt to increase engagement through the gamification of ABMT. *Gamification* refers to the use of digital game elements (eg, points and avatars) in nonentertainment settings [13]. Qualitative [14] and quantitative [15] reviews on gamified cognitive training tasks have found that adding game elements to repetitive tasks improves motivation and engagement. However, Zhang et al [16], in their systematic review that focused specifically on gamified cognitive bias modification interventions for psychiatric disorders (ie, anxiety, affective, and addictive disorders), found that only 2 of the 4 identified studies reported gamified interventions to be effective [17,18], and only 1 study compared a gamified task directly against a nongamified counterpart [19]. This calls for more rigorously designed and theory-driven research in this field.

To date, research has yet to explore the gamified format of ABMT for chronic pain and its potential for the transfer of benefits. To address this gap in the literature, a gamified web-delivered pain ABMT intervention, based on the standard, modified dot-probe task [5], has been developed and augmented with game elements.

### Aims and Hypotheses

The aim of this study is to investigate the effects of a gamified web-delivered ABMT intervention in a sample of adults with chronic musculoskeletal pain via a randomized, double-blind, placebo-controlled trial. To do this, 3 ABMT conditions will be directly compared: nongamified sham control ABMT, nongamified standard ABMT, and gamified ABMT. It is hypothesized that the gamified ABMT condition, relative to both the standard ABMT and control ABMT conditions, will rate more highly on self-reported engagement (ie, task-related engagement, enjoyment, and interest) as well as complete more training sessions and have less dropout. On the basis of the findings from the broader ABMT literature and theoretical considerations [20-22], it is expected that the standard ABMT and gamified ABMT conditions will show a reduction in pain-related attentional biases and interpretation biases after training sessions, with the largest reduction in the gamified ABMT condition. Furthermore, it is expected that both the standard ABMT and gamified ABMT conditions, relative to the control ABMT condition, will show reductions in pain intensity, pain interference, anxiety, and depression scores immediately after training and 1-month later and that these reductions will be greater in the gamified ABMT condition compared with the standard ABMT condition. Finally, it is expected that both the standard ABMT and gamified ABMT conditions, relative to the control ABMT condition, will report global pain-related improvements following training and that these improvements will be larger in the gamified ABMT condition compared with the standard ABMT condition.

## Methods

### Study Design

**Figure 1:**
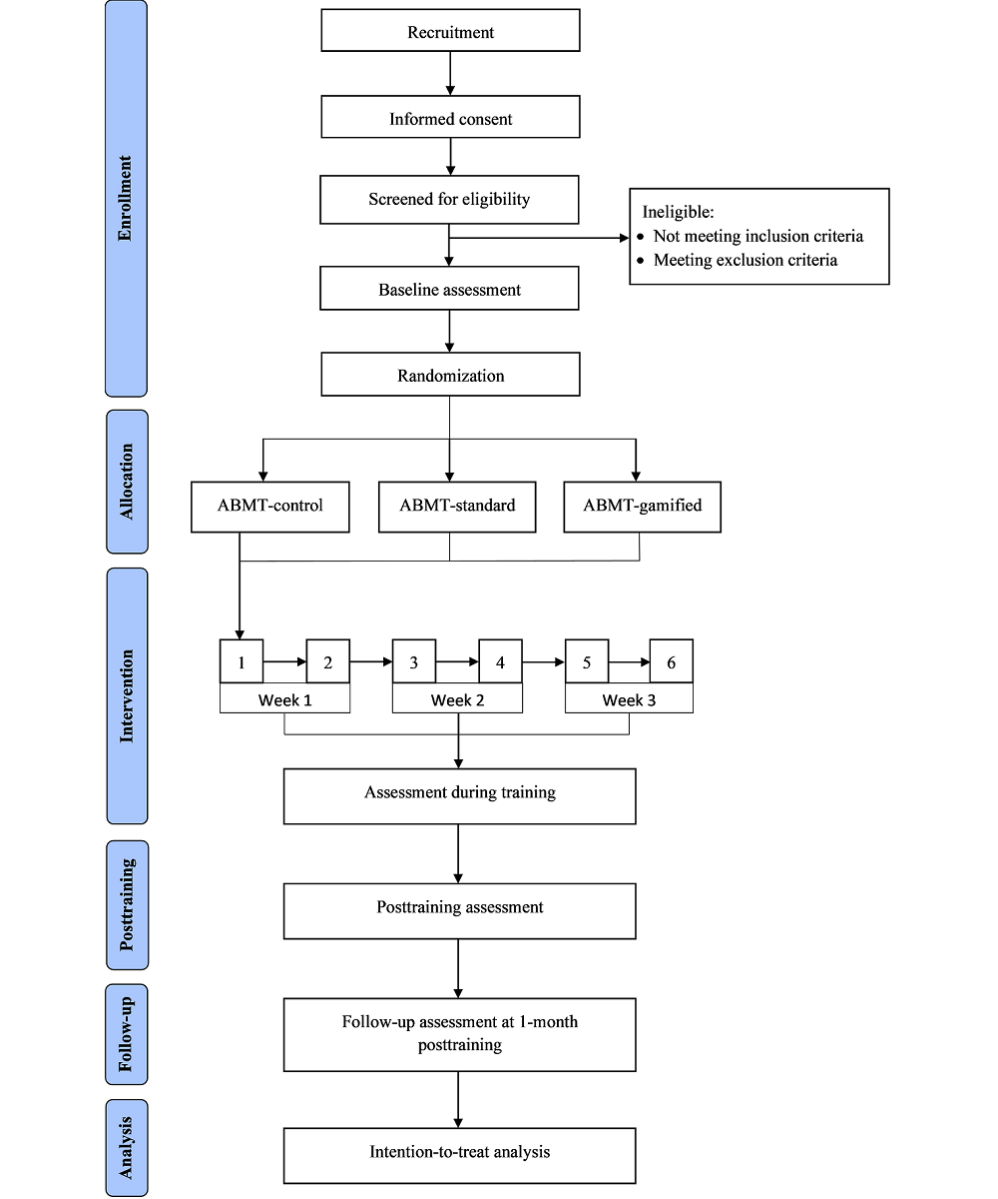
Flow diagram of study protocol. ABMT: attentional bias modification training.

This study is a randomized, double-blind, placebo-controlled, 3-arm, parallel-group trial examining the efficacy of a gamified ABMT for chronic pain delivered over the internet. Adults with chronic musculoskeletal pain will be randomly allocated to 1 of the 3 training conditions: control ABMT, standard ABMT, or gamified ABMT. The control ABMT condition comprises a dot-probe paradigm without training direction, whereas the standard ABMT and gamified ABMT conditions will aim to train attention away from pain-relevant words. Outcome assessments for all training conditions will be conducted via the internet at baseline, during training, immediately after training, and at the 1-month follow-up. Figure 1 shows the trial flow diagram. This study protocol is written in compliance with the SPIRIT (Standard Protocol Items: Recommendations for Interventional Trials) [23] guidelines.

### Study Setting

This trial will take place on the web; that is, all outcome assessments and training sessions will be conducted on the web at the participants’ place of convenience, using their own computers. The study focuses on adults with self-reported chronic musculoskeletal pain, recruited from both clinical (ie, outpatient waiting list) and nonclinical (ie, wider community) settings in Australia.

### Participants

To participate in this study, individuals must be aged ≥18 years; experience chronic musculoskeletal pain, that is, pain in bones, joints, muscles, or related soft tissues (eg, rheumatoid arthritis pain, nonspecific back pain, or fibromyalgia pain); meet the criteria for chronic pain, that is, self-reported pain that lasts or recurs for >3 months [24]; and have normal or corrected to normal vision. Participants will be excluded if they are not native English speakers or not fluent in reading and writing English (as participants’ reaction time [RT] to English words is used as an index of attentional bias to semantically related pain memory networks), have no access to a desktop or laptop computer connected to reliable internet (as the assessments and training sessions are conducted on the web), or are not able or willing to provide informed consent to participate.

### Recruitment and Consent

To optimize the generalizability of the findings, prospective participants will be recruited from a large Australian public hospital outpatient waitlist for pain management (clinical setting) as well as from a wider community (nonclinical setting). Individuals on the hospital outpatient waiting list who have been identified for screening using medical records will be invited to participate through personalized mail correspondence. These individuals may also be approached for recruitment at the introduction to persistent pain management orientation and information sessions held at the hospital every few months. Participants from the wider community will be recruited through university electronic mailing lists, distribution of flyers, social media, and community channels (eg, Facebook advertising, Twitter, LinkedIn, word of mouth, medical practices, and physiotherapy clinics).

All recruitment materials (eg, hospital personalized letters and flyers) will include a survey hyperlink that will direct prospective participants to the necessary study information to decide on participation. This will include information about the research procedures; the voluntary nature of the study, with the freedom to withdraw at any time until the collected data are deidentified (ie, upon completion of the 1-month follow-up survey); the potential risks and benefits of their participation; and whom to contact for questions about the research. All interested participants will be asked to provide informed consent electronically (ie, e-consent) before being taken to the screening questions and then to the first screen of the baseline assessment.

### Patient and Public Involvement

Public members with and without chronic pain have been involved in a prior validation study of pain-related and neutral visual word stimuli sets that will be used in this study (Vermeir JF, White MJ, Johnson D, Crombez G, Van Ryckeghem DML, unpublished data, April 2020). For this study, only patients and members of the public who meet the selection criterion of chronic pain experience will be eligible to participate.

### Randomization, Allocation Concealment, and Blinding

Participants fulfilling eligibility criteria and willing to participate in this study will be randomly allocated to 1 of the 3 training conditions after the baseline assessment. Randomization will be performed before participants are enrolled by an independent person blinded to all processes within the intervention, using a computerized random number generator. A block randomization technique will be used, allowing 6 participants at a time to be randomized in equal proportions to the 3 training arms. The allocation numbers will be stored on a password-protected university database maintained by the same independent person and will be revealed after participants are enrolled and baseline assessments are completed.

Each time a participant completes the baseline survey, an automatic email will be sent to the principal researcher (JFV). After receiving this email, the participant will be allocated to the next number on the list and, consequently, be assigned to a training arm. Researchers and participants will be blinded to group allocation throughout the trial (ie, double-blind study design). Furthermore, as assessments and training will occur on the web, in the absence of the investigators, the outcome data will be blinded. However, it is possible that a researcher could become aware of the participants’ training conditions to support them adequately in the instance of technical problems. However, it is unlikely that this will entail problems of bias allocation or assessment because of the web-based nature of the study.

### Procedure

After informed consent is obtained and participants are eligible, they will complete the web-based baseline assessment (approximately 35 minutes) containing demographic questions as well as questions relating to their general health, current mental health, pain experience, and everyday thoughts and behaviors. At the end of the survey, participants will select their preferred days for training, which will be either (1) Monday and Thursday or (2) Tuesday and Friday. Participants will also be asked to provide an email address so that the research team can send links to the training sessions. Participants will then be randomized into 1 of the 3 groups and be invited by email to start their first training session. This email will include a weblink to the appropriate version of the intervention, as well as instructions on how to download the program.

Training sessions will be performed on the web at the participants’ time and place of convenience (using their own computers), twice a week on a separate pair of days (Monday and Thursday or Tuesday and Friday) for 3 consecutive weeks, totaling 6 sessions. This dosage is based on previous pain ABMT literature, which has shown positive training effects for dosages ranging between 4 and 8 sessions [4,7,8]. It is anticipated that the first and final sessions will take approximately 30 minutes, as it includes cognitive assessment measures, whereas sessions 2 to 5 will take approximately 15 minutes to complete. Participants will be asked to complete the sessions during normal waking hours and within 24 hours of receiving a web link. Each session will start with the same instructions, similar to those of previous research [10], and highlight the need to create a quiet and private environment free from distractions for at least 30 minutes. In total, the participants will be sent 6 session links. Necessary reminders will be sent via email and SMS text messages throughout the study.

After each training session, participants will be asked to rate their experience with the task (<2 minutes). Immediately after completion of the final training session, participants will be invited to the postassessment (approximately 15 minutes), with questions relating to their experience of pain, mood, and other relevant psychological experiences associated with their pain. Finally, 1 month after the end of the training sessions, all participants will receive an email invitation for the follow-up assessment (approximately 10 minutes), with similar questions to that of the postassessment (see *Outcomes and Measures* section for further details).

### Study Program

#### Overview

Participants will be involved in the trial for approximately 2 months, including a 3-week intervention period in which they will be randomized to 1 of the 3 training arms, followed by a 1-month follow-up period. Several strategies will be used to maximize participant retention and follow-up completion. First, we will adopt a web-based completion mode for the surveys and training sessions. Second, participants will receive a personalized email invitation for each training session. These emails will also provide participants the opportunity to ask the research team about any technical difficulties or other obstacles encountered while using the software. Third, we will use a combination of SMS text message and email message reminders according to the participants’ preferences. These reminders will be sent to the participants who do not complete the scheduled session within 24 hours of receiving the weblink. When there is no reaction to the training sessions and reminders after 2 weeks (ie, after 4 training sessions and 4 reminders), the participant will be considered as a dropout. Similarly, participants who fail to complete the follow-up assessments will receive up to 2 emails or SMS text message reminders: one after 24 hours and another one after 48 hours. Finally, the gamified ABMT intervention was developed using gamification features to encourage participants to keep using the program. No incentives will be provided to the participants.

Participant care (eg, rehabilitation program, exercise, cognitive behavioral therapy, and pain medications) concomitant with the ABMT (ie, control ABMT, standard ABMT, or gamified ABMT) will be permitted during the trial. It will be monitored through a *pain-treatment* question that will probe participants’ pain treatments and frequency since the commencement of the study or previous assessment.

#### Task Stimuli

Pain words were chosen as stimuli instead of pictures, as meta-analytic results have shown that biases for pain-related information are larger when using (sensory) pain words than when using pictorial stimuli [3]. Furthermore, a study directly comparing ABMT protocols using words versus facial expressions found that attentional biases changed in the predicted direction on the stimuli presented during the training; however, for those trained on words, training effects also generalized to pictorial stimuli [25]. Finally, words have the advantage of being relatively quick to process, easy to implement, and their physical characteristics (eg, word length) can be tightly controlled [26].

A total of 3 sets of word stimuli will be used. The stimulus set for the practice trials (set 1) will comprise 8 neutral word pairs related to the categories of natural (eg, *log*) and manmade (eg, *pot*) resources. The stimulus set for the experimental trials (set 2) will comprise a set of 8 pain-related words and 8 neutral words. The stimulus set used for the assessment of attentional bias trials (set 3) will comprise 8 different pain-related and neutral word pairs, with the same words presented at pre- and posttraining assessments to investigate the generalization of training effects. Pain words stem from 2 different pain-related categories: sensory (eg, *sharp*) and affective (eg, *agonizing*) pain words. To control for the semantic relatedness of the word set, each pain-related word (eg, *pain*) is matched with a neutral (ie, nonpain) word (eg, *bird*) for length and frequency of use in the English language [27]. Word stimuli in each set will be unique, that is, not replicated in any other set. Each word stimulus will be presented in a black 28-point upper-case Courier New font on a white background. All word stimuli will be taken from a data set of stimulus material, previously created and validated for use in chronic pain samples by the authors of this study (Vermeir JF, White MJ, Johnson D, Crombez G, Van Ryckeghem DML, unpublished data, April 2020). Specifically, in that study, we reviewed the literature on dot-probe studies investigating attentional biases, selected a pool of pain-related and matched neutral words for validation, and then asked participants with and without chronic pain to complete a speeded word categorization paradigm and rate the pain relatedness of a subset of pain words. For this study, we selected sensory and affective pain words that (1) were rated as most relevant to chronic musculoskeletal pain and (2) were categorized the quickest as pain related by adults with chronic pain.

#### Experimental Tasks

#### Overview

Tasks will be programmed and presented using Inquisit 6.4 (Inquisit Web Millisecond software package) on participants’ internet-connected desktop or laptop computers. Participants will be required to download and install the application using a plug-in. At the end of each training session, a data file containing their RT and accuracy scores will be automatically and securely saved to the researcher’s web-based Inquisit account. To account for different screen sizes and ensure consistency in the display of word stimuli across participants, a calibration process will be completed at the start of each session. Participants will be asked to place a credit card (which is universally the same size) on the screen and adjust the length of a horizontal line until it matches the width of the credit card.

**Figure 2:**
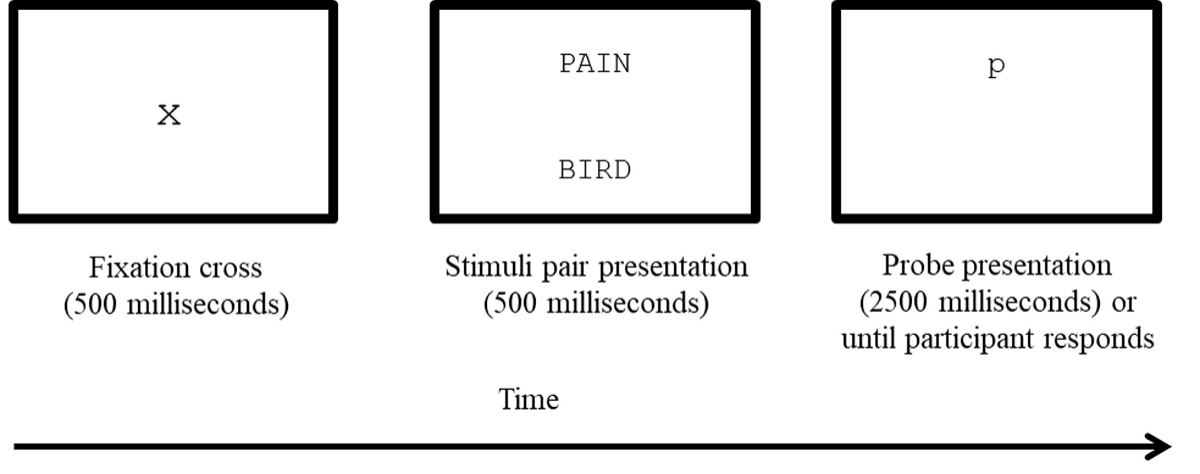
Sample congruent trial from one of the nongamified attentional bias modification training tasks where the dot-probe replaces the top, pain-related word. Stimuli are not presented to scale.

All conditions (standard ABMT, control ABMT, and gamified ABMT) will use a modified version of the dot-probe task [5]. Each task starts with a 500-millisecond duration fixation cross to direct attention to the center of the computer screen (Figure 2). Then, a randomly selected stimulus pair comprising 1 pain-related and 1 neutral word will appear for 500 milliseconds, with 1 word located at the top of the screen and the other at the bottom. The word stimuli will be centered horizontally. Once the pairings disappear, a probe (ie, *p* or *q*) replaces the location of one of the words. Participants will be instructed to determine whether a *p* or a *q* appears and respond as quickly and as accurately as possible by pressing the corresponding keys (ie, *p* key pressed with the right index finger and *q* key pressed with the left index finger) on the computer keyboard. The probe will disappear as soon as a response is recorded or after 2500 milliseconds. The intertrial interval will be 500 milliseconds.

To ensure that participants’ attention is directed toward the center of the screen, several digit trials will be presented [10,28]. In these trials, a random digit number between 1 and 9 will replace the fixation cross for a duration of 150 milliseconds, and participants will be instructed to type the number on the keyboard. The intertrial interval will be 1000 milliseconds after digit trials so that participants can reposition their fingers on the keys. In the context of this study, incongruent trials will be trials where the probe appears in the opposite location previously occupied by the pain-related stimulus, whereas congruent trials will be those where the probe appears in the location previously occupied by the pain-related stimulus.

#### Nongamified Standard ABMT

Each standard ABMT task will start with 1 block of 17 practice trials, comprising 16 neutral stimulus pairs and 1 digit trial. For each correct practice trial, the word *Correct!* will appear on the screen, whereas the word *Incorrect!* will appear for every erroneous response. The training phase will comprise 4 training blocks, each comprising 68 experimental trials (8 [12%] congruent trials; 56 [82%], incongruent trials; and 4 [6%] digit trials), totaling 272 trials and taking approximately 15 minutes to complete. The probe will replace neutral cues in 87.5% (224) of trials and pain cues in 12.5% (32) of trials, thereby training attention away from pain-related cues. This distribution will be used to reduce the obviousness of the probe contingency [29], and participants will not be made aware of it. Word pairs will be randomly presented in each of the 4 possible combinations (probe up and target down, probe down and target up, probe up and target up, and probe down and target down). The selection of a 500-millisecond presentation time for the stimulus pair is guided by previous pain ABMT studies that have shown positive training effects in individuals with chronic pain [4]. Stimuli will be presented in a randomized order across trials and participants, and trials will be intermixed and randomly presented in 4 blocks, with a rest offered between each block of trials.

#### Nongamified Sham Control ABMT

The control ABMT group will be similar to the standard ABMT group except that the probe will appear with equal frequency in the position of the pain-related and neutral words, totaling 272 trials (128 [47%] congruent trials; 128 [47%] incongruent trials; and 16 [6%] digit trials per session) and taking approximately 15 minutes to complete.

#### Gamified ABMT

The implementation of gamification has been listed within the group of complex interventions [30]. These interventions refer to activities that comprise multiple interacting components (eg, intensity and setting) that, when applied to the target population, result in a range of possible outcomes [31]. Therefore, the development of the gamified task followed the Medical Research Council framework for complex interventions [31], using theory, review evidence, and expert involvement. The gamified ABMT task was developed in several steps.

In the first step, a multidisciplinary team with research expertise in the fields of eHealth, cognitive psychology, and gamification discussed the core theories, methods, mode of delivery, implementation strategies, and design requirements. Specifically, rather than developing a completely new ABMT intervention, it was decided to design the gamified ABMT task as a so-called *game-shell* [32]; that is, game elements were added as an additional layer to the standard ABMT task without changing the initial structure. This design allows the original evidence-based ABMT paradigm to remain unchanged and has been frequently used in the gamified cognitive literature [17,32,33].

In step 2, the game elements were selected. This was guided by a qualitative and quantitative review, as well as specific theories. First, we undertook a systematic review and meta-analysis assessing the effectiveness of gamification applied to cognitive training tasks to gain a better understanding of the impact of gamification on cognitive training and identify factors that contribute to the optimal design of such programs [15]. The review identified that typically 5 game elements were used and that achievement and progression-oriented game features (eg, rewards and feedback loops) were commonly implemented in cognitive training tasks. Although the review could not show support for one feature over another (because of a limited number of studies in the subgroups), it provided evidence for the effectiveness of gamification in improving motivation and/or engagement (Hedges *g*=0.72) and synthesized findings into practical guidelines for implementing gamification for cognitive training.

Second, to increase the likelihood of the effectiveness of the intervention design and respond to the call for more theory-driven research on gamification in the field of health [15,34,35], the implementation of game elements in the ABMT procedure was guided by concepts of self-determination theory [36,37] and self-regulation [38]. According to self-determination theory [36,37], which is a well-established theoretical framework within gamification research [34], competence (ie, feeling effective), relatedness (ie, feeling connected to others), and autonomy (ie, feeling a sense of freedom) are the 3 basic psychological needs that determine intrinsic motivation, sustained engagement, and psychological well-being. Previous research has shown that these needs can be addressed by specific elements such as badges, leaderboards, performance graphs, and social competition [39-41]. Another construct that is fundamental to the success of health-related interventions is self-regulation, defined as a dynamic motivational process of setting, pursuing, and maintaining personal goals [42-44]. Self-regulation techniques such as goal setting and self-monitoring can motivate users to engage and sustain in activities, and there is evidence that these techniques can be facilitated in gamification through a range of features such as rewards, goals, levels, and progress bars [45-47].

**Figure 3:**
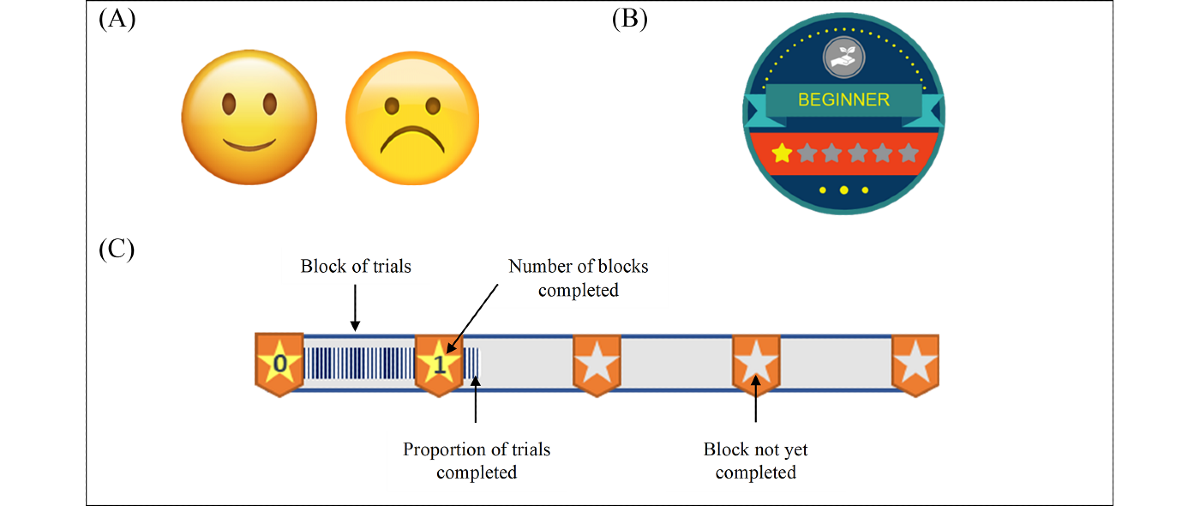
Sample of game elements used in the gamified task. (A) Smiling (left) and frowning (right) emoticons received during practice trials for correct and incorrect responses, respectively. (B) Badge earned at the end of the first training session. (C) Progress bar representing proportion of trials completed in each block and a written indicator reflecting the number of blocks completed.

On the basis of theoretical considerations and the empirical findings discussed in the previous sections, a combination of 5 game elements was incorporated in the gamified task to keep participants motivated and engaged in the sustained and repeated use of the ABMT procedure. The 5 gamification features are briefly described as follows:

1. Clear gamified goal: At the start of each training session, a clear gamified performance goal will be set for the task to earn as many points as possible and receive badges along the way. Goals that are specific and reasonably challenging are the most effective at increasing motivation and task performance [48] and are likely to increase the satisfaction of the need for competence [40].
2. Feedback loops: During the practice phase, immediate gamified feedback will be provided to help facilitate self-monitoring [46,49] and feelings of competence [40]. For each correct practice trial, the word *Correct!* and a smiling emoticon will appear on the screen, whereas the word *Incorrect!* and a frowning emoticon will occur in every incorrect practice trial (Figure 3).
3. Task-related progress: During the training phase, a constantly visible progress bar at the top of the screen will indicate the proportion of trials remaining in each block, and a written indicator will reflect the number of blocks completed (Figure 3). These gamification features can facilitate self-tracking and motivate participants toward the attainment of goals [45,46] and fulfill their desire for competence [39].
4. Rewards: Collectible points and badges were implemented to facilitate participant goal setting [45,46] and satisfy their innate psychological needs for competence, autonomy, and relatedness [40]. Specifically, between blocks of trials, participants will receive feedback about their performance in the form of points, calculated for each block of trials (1 point is earned for each correct trial; a maximum of 68 points can be earned per block). We choose to provide feedback after each block of trials rather than after each trial to ensure that the flow of training is uninterrupted. At the end of each training session, the participants will also be rewarded with a badge (Figure 3). There are 6 different badges, each of which has a number of stars on it corresponding to the number of sessions completed.
5. Sound effect (with rewards): To enhance motivation, the task incorporates a pleasant auditory and visual reward in the form of a firework. To ensure that all participants in the gamified ABMT condition are exposed to the same type of game elements, everyone will experience the fireworks after the first block of trials. However, for subsequent blocks of trials, only those who obtain at least 60% (41/68 trials) accuracy will experience the fireworks. This latter criterion involves an element of uncertainty that can further increase motivation.

In step 3, a gamified prototype for the experimental condition was created collaboratively by the authors of this paper, who are experts in the field of gamification and cognitive psychology, and programmed using Inquisit 6.4.

In the last step, all the intervention materials and tasks (nongamified and gamified) were piloted internally by members of the research team to ensure that the program was feasible to deliver over the internet to the target sample. Following discussions, minor refinements were proposed and made to the gamified intervention. For example, the width and height of the progress bar were adjusted to make the visibility of the progression more noticeable.

### Outcomes and Measures

#### Overview

Tasks will be presented using Inquisit 6.4 on participants’ internet-connected computers, and questionnaires will be administered using the web-based system Qualtrics (Provo), except for self-reported engagement, which will be administered using Inquisit 6.4. Assessments for all training conditions will be conducted at baseline, during training, immediately after training, and at the 1-month follow-up. A list of all outcomes, measurement instruments, and corresponding time points is presented in Table 1.

**Table 1:** Study outcome measures by assessment time point.

Outcome and variable		Measure	Assessment time point			
			Baseline	During training	Posttraining	1-month follow-up
**Baseline data**						
	Eligibility screening	Self-report questions	✓			
	Demographic information	Self-report questions	✓			
	**Pain experience information**					
		Self-report questions	✓			
		GCPS^a^	✓			
**Primary outcomes**						
	Pain intensity	PROMIS^b^ Pain Intensity 3a	✓		✓	✓
	Pain interference	PROMIS Pain Interference 8a	✓		✓	✓
	**Self-report engagement**					
		1 self-report question		✓	✓	
		IMI^c^ (interest and enjoyment)			✓	
	**Behavioral engagement**					
		Intervention attrition rates		✓	✓	
		Completion rates		✓	✓	
**Secondary outcomes**						
	Attentional bias	Dot-probe task	✓		✓	
	Anxiety	PROMIS Anxiety 8a	✓		✓	✓
	Depression	PROMIS Depression 8b	✓		✓	✓
	Perceived improvement	PGIC^d^			✓	✓
	Interpretation bias	Interpretation bias task	✓		✓	
**Additional measures**						
	Attentional control	ACS^e^	✓		✓	
	Personality characteristics	BIS and BAS^f^ scales	✓			
	Pain-related worrying	PCS^g^	✓			
	Pain-treatment information	Self-report questions			✓	✓
	Validity check	Instructional question	✓		✓	✓
	Manipulation check	1 self-report question			✓	

^a^GCPS: Graded Chronic Pain Scale.

^b^PROMIS: Patient-Reported Outcomes Measurement Information System.

^c^IMI: Intrinsic Motivation Inventory.

^d^PGIC: Patient Global Impression of Change.

^e^ACS: Attentional Control Scale.

^f^BIS and BAS: Behavioral Inhibition System and Behavioral Activation System Scales.

^g^PCS: Pain Catastrophizing Scale.

#### Baseline Information

At baseline, participants will report on demographic information pertaining to age, gender, first language, country of birth, country of residence, postcode of current home address, ethnicity, marital status, employment status, education level, and hand preference. Participants will also report on the type of computer (eg, laptop), screen size and keyboard (eg, QWERTY) they are using, their health in general, whether they currently have a mental health condition (eg, depression), and on their pain experience information, including current pain problems, formal diagnosis of the pain condition (ie, by a physician), duration of primary pain condition, body locations where they experience pain, area of the body that hurts the most, how the primary pain condition began (eg, postsurgical), current treatment for the pain problem (eg, physiotherapy), and frequency of health care use.

To provide additional information characterizing participants’ overall pain severity, participants will complete the Graded Chronic Pain Scale (GCPS) [50]. The GCPS is a 7-item self-report instrument designed to assess 2 dimensions of chronic pain severity (pain intensity and pain-related disability) in the general population and in primary health care settings. The scale measures the presence of chronic pain in the past 6 months, and all items, except for the number of days disabled, are scored on an 11-point Likert scale, with responses ranging from 0 to 10. Subscale scores (ie, characteristic pain intensity, disability score, and disability points) for the 2 dimensions are combined to calculate a chronic pain grade that allows individuals with chronic pain to be classified into 1 of 5 hierarchical categories: grades 0 (*no pain problem*) to 4 (*high disability-high intensity*). The GCPS has been found to have acceptable to excellent internal consistency, with a Cronbach α ranging from .74 to .91 [50,51]. Dunn et al [52] found that the test–retest reliability after a 2-week interval was good, with a weighted Cohen κ of 0.81.

#### Primary Outcome Measures

##### Pain Intensity

Pain intensity will be assessed using the Patient-Reported Outcomes Measurement Information System (PROMIS) Pain Intensity–Short Form 3a (v1.0; 3 items) [53]. The first 2 items assess pain intensity using a 7-day recall period and are rated on a 5-point Likert scale ranging from 1 (*no pain*) to 5 (*very severe*). The last item asks participants to rate their level of pain *right now* and is rated on a 5-point Likert scale that ranges from 1 (*no pain*) to 5 (*very severe*). Raw score totals are transformed to T-score metrics using the PROMIS conversion tables, such that the average score for the general population is 50 and the SD 10. Higher T-scores represent worse pain. PROMIS Pain Intensity has been shown to be valid for assessing pain in various settings [54].

##### Pain Interference

The impact of pain on daily life will be assessed using the PROMIS Pain Interference–Short Form 8a (version 1.0; 8 items) [53]. The items have a 7-day time frame and are rated on a 5-point Likert scale ranging from 1 (*not at all*) to 5 (*very much*). Raw score totals are transformed to T-score metrics using the PROMIS conversion tables, such that the average score for the general population is 50 and the SD 10. Higher T-scores represent greater pain interference. PROMIS Pain Interference has been assessed and validated in both general and clinical populations [54,55].

##### Engagement

Participants’ experiences of engagement will be assessed using 2 self-report measures. Task-related engagement will be measured after each training session with a single-item question: *How engaging was this session?* The item is rated on a 10-point Likert scale, ranging from 1 (*not at all*) to 10 (*very much*), with a higher score indicating greater engagement. Task-related interest and enjoyment will be assessed using the Intrinsic Motivation Inventory—Interest and Enjoyment subscale [56-58]. The subscale comprises 7 items, which are scored on a 7-point Likert scale ranging from 1 (*not at all*) to 7 (*very true*), with higher scores representing higher levels of interest and enjoyment. The reliability and validity of this subscale have been established in previous research [59,60].

Nonuse intervention attrition [61] (ie, the proportion of participants who discontinue using the intervention at each training session) and completion rates (ie, the proportion of sessions, out of 6, that each participant completes during the training period) will be used as objective behavioral measures of engagement.

#### Secondary Outcome Measures

##### Measure of Attentional Bias for Pain

Attentional biases will be measured using the standard dot-probe paradigm [62]. This task is similar to the one used during the experimental phase, except that the probe replaces each of the words in each pair with equal frequency. Before the assessment blocks, participants will complete a block of 17 practice trials, comprising 16 neutral stimulus pairs and 1 digit trial. Stimuli will be presented in a randomized order across trials and participants, and trials will be intermixed and randomly presented in 2 blocks, with a rest offered between the blocks. Each block will comprise 68 trials (32 [47%] congruent trials; 32 [47%] incongruent trials; 4 [6%] digit trials), totaling 136 trials. Consistent with previous research [10,28], practice trials, digit trials, incorrect trials, and responses <200 milliseconds or >1000 milliseconds will be excluded from the calculation of mean RTs. An attentional bias index will be calculated using the following formula: mean RT of incongruent trials–mean RT of congruent trials. Positive scores will be indicative of an attentional bias toward pain-related stimuli, whereas negative scores will reflect an attentional bias toward neutral stimuli.

##### Anxiety and Depression

Negative affect will be assessed with 2 PROMIS measures comprising PROMIS Anxiety 8a (version 1.0; 8 items) and PROMIS Depression 8b (version 1.0; 8 items) [53]. The items have a 7-day period and are rated on a 5-point Likert scale ranging from 1 (*never*) to 5 (*always*). The raw score totals on each scale will be transformed to T-score metrics using the PROMIS conversion tables, such that the average score for the general population is 50 and the SD 10. Higher T-scores represent greater symptoms of anxiety or depression. The 2 PROMIS measures have demonstrated excellent psychometric properties in both population-based [63] and clinical samples [64,65].

##### Perceived Improvement

Participants’ perception of overall pain-related improvement following training will be assessed using the Patient Global Impression of Change (PGIC) scale [66]. This measure comprises a single item rated on a 7-point Likert scale, ranging from 1 (*very much improved*) to 7 (*very much worse*), with *no change* in the middle of the scale (4). For descriptive purposes, participants will be classified into 3 categories according to the PGIC score: disease deterioration (0-3 points), stable disease (4 points), or disease improvement (5-7 points) since the start of the program [67]. The PGIC has been recommended by the Initiative on Methods, Measurement, and Pain Assessment in Clinical Trials [68] and is widely used in chronic pain research [67,69].

##### Measure of Interpretation Bias for Pain

Interpretation bias will be measured using an adapted version of the computerized interpretation bias task [70], which contains 16 vignettes that describe 8 ambiguous situations that may be interpreted as relating to bodily threat or pain and 8 ambiguous social situations. Vignettes were adapted to reflect events that may occur in the workplace, home, or during an adult’s everyday life. Participants will be instructed to imagine themselves in the situation, and after reading each ambiguous scenario, they will be offered end words that resolve the situation in a benign or negative manner. Participants will then rate whether each resolution came to their mind on a scale of 1 (*does not pop into my mind*) to 5 (*definitely pops into my mind*) and select the interpretation (word) that most easily popped into their head. Next, participants will be presented with the same scenarios again; however, this time, they will be asked to rate the likelihood that each resolution would actually happen in that situation on a scale of 1 (*not likely*) to 5 (*very likely*). Finally, participants will select the word that they believe is most likely to end the sentence. All items and interpretations will be presented in a fixed random order to ensure all participants view the same order of items and response choices. Studies using this task have found evidence of interpretation bias in relation to pain in individuals with chronic pain [71].

#### Additional Measures

##### Measure of Attentional Control

Attentional control will be assessed using the Attentional Control Scale (ACS). The ACS [72] is a 20-item self-report questionnaire used to measure attention focusing and attention shifting. The questionnaire is adapted by including a 7-day time frame for the items. Items are scored on a 4-point Likert scale from 1 (*almost never*) to 4 (*always*), with scores ranging from 20 to 80. Higher scores indicate a better ability to direct and maintain attention. The ACS has been found to have good reliability, with a Cronbach α of .81 [10] and good concurrent validity [72].

##### Personality Characteristics

Personality traits will be measured using the Behavioral Inhibition System (BIS) and Behavioral Activation System (BAS) scales [73], a 20-item self-report questionnaire that measures trait sensitivity levels of the BIS (punishment; 7 items) and BAS (reward; 13 items). The scales are scored on a 4-point Likert scale ranging from 1 (*strongly disagree*) to 4 (*strongly agree*). Meyer et al [74] found that internal consistency ranged from acceptable to good for the BIS and BAS scales. Test–retest reliability over 2 months was acceptable for both scales [73].

##### Pain-Related Worrying

Participants’ pain-related worrying [75] will be assessed using the Pain Catastrophizing Scale (PCS) [76]. The PCS is a 13-item self-report measure that evaluates 3 subscales: rumination, magnification, and helplessness. Using a 5-point Likert scale, ranging from 0 (*not at all*) to 4 (*all the time*), participants will be asked to recall past painful experiences and indicate the extent to which 13 thoughts or feelings are associated with these experiences. The 3 subscale scores are summed to provide a total score for pain catastrophizing, which ranges from 0 (*low degree of catastrophizing*) to 52 (*high degree of catastrophizing*). The PCS has been found to have good validity and reliability for individuals with chronic pain [76,77].

##### Pain-Treatment Information

At the posttraining and 1-month follow-up assessments, there will be a question that probes participants’ pain treatments and frequency of health care use since the commencement of the study or previous assessment.

##### Manipulation Check

At the posttraining assessment, there will be a manipulation check question asking participants which training condition they believe they had received (ABMT or sham training).

##### Validity Check

As recommended by Oppenheimer et al [78], instructional questions will be included in the pre- and posttraining surveys (eg, please select *5=Always*) to identify careless responding patterns. Participants will be excluded if they answer all instructional questions incorrectly.

### Data Management and Monitoring

Owing to the minimal risks associated with study participation, it is not necessary to implement an independent data and safety monitoring board. The research team will be responsible for monitoring and data management and will meet regularly to manage the protocol, monitor recruitment, and deal with any adverse events. The reporting of this study will be conducted according to the CONSORT (Consolidated Standards of Reporting Trials) statement guidelines [79].

All data will be collected via the web using Qualtrics (Provo) for survey responses and Inquisit 6.4 for task data and self-reported engagement responses, and temporarily stored on these servers. Data safety and security measures have been considered, including restricted access to the research team, password protection, firewall, and virus protection. Furthermore, each participant will be assigned a unique participant code (blinded to the group the participant has been assigned to) and will be asked to self-generate an ID code (instead of participants’ personal information). Thus, this coded data may be reidentifiable during the research but will be deidentified upon completion of the study. An electronic, password-locked master file will be created that matches the unique participant code to their self-generated code to ensure participants are allocated to the correct experimental group and that the survey and task data match across the different time points. All data will be stored on a secure password-protected university server and accessed only by the research team. Upon completion of the project, electronic research data will be deposited in the university’s research data storage system and retained for a minimum of 15 years. The final coding scheme for outcome measures will be available from the authors upon request.

On the basis of previous similar trials, no adverse events are expected [10]. A small amount of fatigue and some mild discomfort during the training task may be experienced. This will be managed by providing participants with enough rest periods between blocks of trials. If the research team becomes aware of any harm or other adverse events, it will be documented and reported appropriately. The research team will also manage any risks and recommend participants to liaise with relevant services, such as psychological assistance, if appropriate. The study will be stopped if evidence emerges that participants can come to harm because of the ABMT intervention.

### Sample Size Estimation

To the best of our knowledge, there are no similar published studies on gamified pain ABMT or ABMT for adults with chronic pain that directly compare 3 groups (ie, control ABMT, standard ABMT, and gamified ABMT); therefore, there is no previous effect size on which to base a sample size estimation. As such, a minimum sample size of 30 per training group is planned on the basis that this exceeds the sample size determined by several similar pain ABMT and gamified training studies [7,10,80]. Sharpe et al [7] determined that a sample size of 12 per group was enough to achieve 82% power with a significance level of .05 for their study of ABMT in adults with chronic pain that compared 2 groups (ABMT vs placebo), for which a medium effect size (Cohen *d*=0.45) on pain interference was found in the intervention group (ABMT: n=22; placebo: n=12). Heathcote et al [10] determined a sample size of 20 per group for their study of ABMT in adolescents with chronic pain that compared 3 groups (ie, ABMT, placebo, and waitlist). Boendermaker et al [80] observed in their study, which involved a sample size of <20 per group, that gamified cognitive bias modification training (for alcohol problems) had a positive impact on motivation to train compared with regular training. Considering attrition rates of previous trials in chronic pain treatment [81] and given the 1-month follow-up measurement, a dropout rate of approximately 30% is expected for the current trial. Therefore, a total target sample size of 120 participants (40 participants per group) will be sought. To help achieve adequate participant enrollment, we developed engaging recruitment materials and selected multiple channels for delivery.

### Statistical Methods

#### Overview

Statistical analyses will be conducted after data collection is completed using the SPSS version 27.0 or later (IBM Corp). Continuous data will be presented as means and SDs, whereas categorical data will be presented as frequencies and percentages. Analyses will follow the intention-to-treat principle and will include all randomized participants who successfully complete at least one training session (ie, minimum threshold exposure to ABMT). To determine whether there are any pretraining differences between the training conditions on demographic variables and baseline characteristics, a series of analyses will be performed. The rates of and reasons for missing data will be reported. To manage missing data, the study will attempt to follow-up on all randomized participants (even if they withdraw from the trial) and, where appropriate, use multilevel modeling for repeated measure data analyses as it allows the incorporation of all available data. Significance for all statistical tests will be set at *P*<.05 (2-tailed). Effect sizes will be presented by its most appropriate effect size (and 95% CI), as described by Lakens [82], with a preference for Cohen *d* where possible [83]. No interim analyses of the trial outcome data are planned.

#### Symptom Measures

To determine symptom changes (ie, pain intensity, pain interference, anxiety, and depression) in the different training conditions, multilevel modeling analyses will be conducted. For each model, the time point (level 1 units) will be nested within participants (level 2 units). The variable time for each symptom measure will have 3 levels (ie, pretraining, posttraining, and 1-month follow-up), with pretraining as the reference point. The control group (control ABMT) will serve as a reference group for comparisons between the groups over time. A model-building procedure [84] will be used to build the most parsimonious model to test the hypotheses, using the Akaike information criterion (AIC) to identify the most appropriate model. All models will be computed using maximum likelihood estimation.

#### Engagement Measures

To examine changes in task-related engagement in different training conditions, a multilevel modeling analysis will be conducted. The time point (level 1 units) will be nested within participants (level 2 units). The variable time will have 6 levels (ie, after sessions 1, 2, 3, 4, 5, and 6). A model-building procedure [84] will be used to build the most parsimonious model to test the hypotheses, using the AIC to identify the most appropriate model. The model will be computed using maximum likelihood estimation. To analyze the impact of gamification on interest and enjoyment, a 1-way analysis of variance will be performed. Missing values will be handled using multiple imputations.

Regarding behavioral engagement, Kaplan-Meier survival curves [85] will be calculated to assess the time at which attrition occurred in each training condition and compared statistically using a log-rank test. Participants will be classified as noncompleters if they do not complete all 6 training sessions. Finally, a 1-way analysis of variance will be performed to determine whether there are differences in the mean number of sessions completed between the training conditions.

#### Cognitive Measures

To determine changes in attentional bias and interpretation bias in the different training groups, multilevel modeling analyses will be conducted. For each model, the time point (level 1 unit) will be nested within participants (level 2 units). The variable time for each cognitive measure will have 2 levels (ie, pre- and posttraining), with pretraining as the reference point. The control group (control ABMT) will serve as a reference group for comparisons between the groups over time. A model-building procedure [84] will be used to build the most parsimonious model to test the hypotheses, using the AIC to identify the most appropriate model. All models will be computed using maximum likelihood estimation. In addition, Pearson correlations will assess the relationship between changes in attentional bias magnitude from pre- to posttraining and changes in scores on symptom measures (ie, pain intensity, pain interference, anxiety, and depression).

#### Perceived Improvements

To determine changes in perceived improvement in the different training groups, a multilevel modeling analysis will be conducted. The time point (level 1 units) will be nested within participants (level 2 units). The variable time will have 2 levels (ie, posttraining and 1-month follow-up), with posttraining as the reference point. The control group (control ABMT) will serve as a reference group for comparisons between the groups over time. A model-building procedure [84] will be used to build the most parsimonious model to test the hypothesis, using the AIC to identify the most appropriate model. The model will be computed using maximum likelihood estimation.

#### Exploratory Analyses and Subgroup Analyses

Additional exploratory and subgroup analyses will be performed to explore the role of engagement metrics (ie, number of training sessions completed) and individual differences (ie, attentional control, pain-related worrying, personality characteristics, and recruitment setting [clinical vs nonclinical]) in the impact of training conditions on the primary outcomes. These analyses will be conducted using appropriate statistics and are subject to the final sample size and power.

### Ethics and Dissemination

This trial has been approved by the human research ethics committees of the Royal Brisbane and Women’s Hospital and Queensland University of Technology and registered on the Australian New Zealand Clinical Trials Registry, ACTRN12620000803998, version 1.0, approved on August 10, 2020. All participants will provide informed consent electronically before inclusion in the trial. Of note is that participants will not be provided information about the 3 training conditions (ie, control ABMT, standard ABMT, and gamified ABMT). They will be informed that the aim of the study is to test whether a novel psychological program can reduce chronic pain and improve pain-related health outcomes and that they will be randomly assigned to either receive the intervention or complete a similar task (the control group). In doing so, participants will remain blinded to their allocation, as it would be easy to realize whether they are in the *active* gamified group or not (and noting that the control group does not include game-like features). Participants will be debriefed at the conclusion of their study involvement. Participants who received the intervention will no longer have access to the computerized task. Participants in the control ABMT condition will be offered the opportunity to perform the standard ABMT training. No data will be collected, used, or analyzed during that time. At the completion of the final session, the participants will no longer have access to the intervention.

Any modifications to the study protocol will be recorded and communicated with the human research ethics committees and the clinical trial registry. The final data set will be accessible to approved members of the research team. The results of this trial will be reported in the form of a doctoral research thesis (for JFV) and published in a peer-reviewed journal and presented at conferences. No professional writers will be used. A lay summary of the outcomes and results of the study will be made available to the participants.

## Results

Ethics approval for this study was granted by the human research ethics committees of the Royal Brisbane and Women’s Hospital in April 2020 and Queensland University of Technology in September 2020. Study recruitment commenced in August 2021 and is ongoing. Data collection and analysis are expected to be concluded by October 2022 and January 2023, respectively. The results of this study are expected to be published in mid-2023.

## Discussion

### Study Contributions

This protocol describes a randomized, double-blind, placebo-controlled trial aimed at evaluating the effects of a gamified web-delivered ABMT intervention on pain intensity, pain-related outcomes, cognitive biases, behavioral and self-reported engagement, and perceived improvement in a sample of adults with chronic musculoskeletal pain. The use of gamification in such programs may have the potential to keep participants engaged and motivated in the sustained and repeated use of the task as well as increase retention.

To our knowledge, this is the first study to evaluate the effects of gamification techniques in a pain ABMT intervention. This study will provide important information on the potential therapeutic benefits of gamified pain ABMT programs and shed light on the motivational influences of certain game elements in the context of pain. If results support its effectiveness, this novel, web-delivered, easy-to-administer cognitive program could open new avenues for alleviation of pain suffering, thereby improving the quality of life for these individuals and their families. This is especially significant considering that chronic pain affects between 19% and 31% of the population worldwide [86-88]. Finally, the expected findings will provide novel contributions to the knowledge base and understanding of chronic pain. This, in turn, may open new directions for therapeutic interventions.

### Strengths and Limitations

This study has significant strengths. First, findings from the 3-arm randomized controlled trial design, with the inclusion of a nongamified version of the intervention, will allow us to determine whether the addition of game elements leads to increased motivation, engagement, and intervention efficacy. Further strengths of the study include (1) the rigorous, well-designed, and prespecified study protocol that has been reported as per SPIRIT [23] guidelines and preregistered on a clinical trial registry; (2) the use of an empirically supported set of pain-relevant word stimuli; (3) the inclusion of self-report and behavioral measures of engagement as a primary outcome; (4) the large chronic pain sample drawn from clinical and nonclinical settings; and (5) the inclusion of a 1-month follow-up assessment.

Despite its strengths, this study also has limitations. First, although restricting our sample to individuals with chronic musculoskeletal pain strengthens the methodology of the study, this may limit the generalizability of the results to other types of chronic pain conditions. However, at this early stage in investigating the impact of game elements on ABMT, we believe that the advantages of researching a homogeneous sample outweigh the benefits of a heterogeneous sample. Second, the current ABMT approach provides limited control over the environment under which ABMT will be completed, which could potentially affect the results. However, again, the use of a web-based delivery mode was an informed choice. There is an increasing need and demand in society for remote delivery and access to health treatments, which has been highlighted by the current COVID-19 pandemic [89] and isolation environment and is also of particular benefit to those living in regional and remote areas. With the move to web-based administration and the resulting reduction in face-to-face interpersonal interaction, there may arguably be an even greater need for these interventions to include features such as gamification to facilitate engagement and continued motivation to self-administer such tasks. Finally, the use of the dot-probe task will only allow us to provide a static snapshot of the dynamic attentional process that unfolds over time [6]. Currently, web-based measures investigating how attentional dynamics resolve over time are not yet widely accessible; therefore, we opted for the dot-probe assessment paradigm, as it is frequently used in web-based attentional bias research [9].

## Abbreviations

ABMT: attentional bias modification training
ACS: Attentional Control Scale
AIC: Akaike information criterion
BAS: Behavioral Activation System
BIS: Behavioral Inhibition System
CONSORT: Consolidated Standards of Reporting Trials
GCPS: Graded Chronic Pain Scale
PCS: Pain Catastrophizing Scale
PGIC: Patient Global Impression of Change
PROMIS: Patient-Reported Outcomes Measurement Information System
RT: reaction time
SPIRIT: Standard Protocol Items: Recommendations for Interventional Trials
